# Health Locus of Control in Patients With Graves-Basedow Disease and Hashimoto Disease and Their Acceptance of Illness

**DOI:** 10.5812/ijem.3932

**Published:** 2012-06-30

**Authors:** Malgorzata Anna Basinska, Anna Andruszkiewicz

**Affiliations:** 1 Kazimierz Wielki University in Bydgoszcz, Bydgoszcz, Poland; 2 Nicolaus Copernicus University in Torun, Torun, Poland

**Keywords:** Graves Disease, Hashimoto Disease

## Abstract

**Background:**

Adaptation to a chronic somatic disease depends on a variety of factors, including belief in health locus of control.

**Objectives:**

Correlation between health locus of control and illness acceptance in patients with Graves-Basedow and Hashimoto diseases as well as correlation between health locus of control, illness acceptance, sex, and age.

**Patients and Methods:**

Three methods were applied: Multidimensional Health Locus of Control Scale by K.A. Wallston, B.S. Wallston and R. DeVellis; the Acceptance of Illness Scale by B.J. Felton, T.A. Revenson, and G.A. Hinrichsena; and a personal questionnaire. Two groups were subject to the research: 68 patients with Graves-Basedow disease and 54 patients with Hashimoto disease.

**Results:**

Patients with Graves-Basedow disease, women above all, have their health locus of control in other persons (P = 0,001) and are less inclined to accept their illness (P = 0,005) when compared to patients with Hashimoto disease. A statistically significant correlation occurred between the age of patients and external (i.e., in other persons) health locus of control.

**Conclusions:**

Beliefs in health locus of control and type of illness in female patient group are predictors of illness acceptance (P = 0,0009).

## 1. Background

Beliefs are cognitive elements of individual’s personality. They determine what we consider as true or false; owing to those beliefs we can answer the questions about other people, the world that surrounds us, or situations in which we can find ourselves. Each of us has different beliefs and accompanying emotions that lead to different conclusions. They are important elements of personality by which we are able to take our decisions, including decisions on our health (1).

A chronic somatic disease makes an individual face with a number of new challenges, and in most cases patients approach their existing life by creating a subjective representation of the illness, the so-called illness representation. Illness representation and individual’s behaviour are affected, inter alia, by beliefs concerning locus of control (LOC), and in particular, health locus of control (1, 2).

If an individual perceives an event subject to control of his/her actions or long-lasting features, then we refer to as internal locus of control. If the result is perceived by a person as an effect of luck, chance, destiny, or of being controlled by others, then we refer to as external locus of control (2).

The concept of health locus of control, created by the Wallstons (3, 4), is based on Rotter’s theory and the holistic perception of health and illness. Health control may be described as a belief owing to which an individual believes s/he affects his/her own internal state and behaviour as well as environment, thus obtains desired results (3). This control performs a vital role in the importance assessment of stimuli and expectations and their influences on human behaviour (5).

Individuals with internal LOC are more responsible for their health, and for recovery and rehabilitation. In comparison to individuals with external LOC, they are more prone to undertaking efforts in order to feel well. Yet, this occurs only when an individual places health high in hierarchy of values (4). Persons having high inner containment are more prone to expanding their health-related knowledge (6) and being more treatment-compliant (7). The beliefs in internal locus of control positively correlate with applying more active ways of coping with stress (8). In general, they adapt more positively to the illness in terms of psychological and physical conditions (9).

Generally, external health locus of control is related to having chronic illnesses, with negative emotions such as depression, hostility, and anxiety, and with physical symptoms. Individuals with external locus of control apply passive avoidance ways of coping more frequently (8). External LOC may affect somatic health condition through its relationship to psychological wellness, on which patient does not contribute to undertake health related behaviours (10, 11).

An emotional indicator to patient’s behaviour and his or her adaptation to an illness is the degree of illness acceptance reflected in minor intensification of negative reactions and emotions related to currently experienced illness, and in admission to illness related constraints. Illness acceptance refers to admission to such constraints and insight to resultant losses (12). Patients achieved illness acceptance react more moderately to experienced health related difficulties than patients unable to reconcile with undesirable health conditions. Illness acceptance, in general, results in better adaptation to a situation. Persons reconciled with their own health, though involved in chronic diseases, think over more realistically and do not expect outcomes beyond achievable treatment results (13).

Latest researches, although not so explicit in all conclusions, reveal that illness acceptance by patients affects positively on quality of their general health and life conditions. In a group of patients with systemic lupus erythematosus, illness acceptance together with optimistic attitudes appeared to be a strong predictor of life quality (14). In patients with rheumatoid arthritis, decrease in illness acceptance together with increase in negative attitudes was observed in alignment with progress of disability (15). In a group of patients with Graves-Basedow disease a correlation was observed between intensity level of dispositional optimism and acceptance of the illness (16). In a group of patients suffering from diabetes those who accepted their illness were able to control their metabolism more effectively (15, 17).

Present results of studies indicate that illness acceptance is not an expression of weakness and resignation, but rather it stems from self-confidence of the person thereby accepts and reconciles with everything that is out of his/her control; in consequence it helps him/her to live with and tolerate well the disease. Lack of illness acceptance may result in noncompliance, hence delay of healing process or emerge of complications may sometimes occur (13).

Graves-Basedow and Hashimoto diseases are examples of chronic disorders requir systematic and regular control of patient’s own health and acceptance inevitable outcomes. They are categorised as autoimmune diseases caused by cell- and antibody mediated processes against specific thyroid cells in each disorder. The ethio-pathogenesis of these diseases is complex and not yet full recognised. The diseases usually develop rapidly and trigger factors like psychological or physical shocks, or a virus infection may frequently proceed the onset (18). Contemporary medical knowledge still has not achieved full effective treatments for these disorders and thereby patiens’ entire life are often involved.

Patients with chronic endocrine diseases require to be consulted about their daily routine health related activities and warranted to reconcile with untreatable and inevitable conditions that may occur.

## 2. Objectives

As no studies in the past were performed on patients with autoimmune thyroid disein the context of illness acceptance and its correlation with health locus of control, our research were carried out. The following research questions were proposed:

1. What is specificity of health locus of control and illness acceptance in patients with Graves-Basedow and Hashimoto diseases?

2. What is the relationship between health locus of control and illness acceptance with patients’ age?

3. What is the relationship between health locus of control and illness acceptance in patients with Graves-Basedow and Hashimoto diseases?

## 3. Patients and Methods

The research was conducted individually among patients of Department and Clinic of Endocrinology. The consent of Commission of Ethics to perform the research was obtained. Participation in the research was voluntary; patients expressed their consents in writing and, filled up questionnaires.

68 persons with Graves-Basedow disease (group G-B) and 54 patients with Hashimoto disease (group H) participated in the research. In both groups, number of participated women was more than participated men (in group G-B there was 55 women and 13 men, i.e. 81 % and 19 %, respectively; in group H there was 46 women and 8 men, i.e. 85 % and 15 %, respectively). This is because of higher incidence and prevalence of both diseases in women rather than in men. Due to substantial diversity in patients’ sex, all analyses could not be executed, thereby research results should be interpreted rather carefully.

Mean values of age for group G-B and group H were 47.28 (SD = 11.81; from 20 to 70 years) and 48.5 years (SD = 13.94; from 19 to 74 years), respectively. In regard to educational level, the largest groups consisted of patients with secondary (n = 54; 44 %) and vocational (n = 32; 26 %) education, and the smallest groups with primary (n = 10; 8 %) and university (n = 26; 22 %) education.

Three research methods were applied: the Multidimensional Health Locus of Control (MHLC-B) by K.A. Wallston, et al (19, 20), the Acceptance of Illness Scale (AIS) by B. Felton, et al (15) and a personal questionnaire that covered basic information of the patient.

The MHLC-B scale (Multidimensional Health Locus of Control) consists of 18 statements on beliefs concerning generalised expectations in three dimensions of health locus of control, i.e.: internal (I), influence of other people (I), and influence of chance (P). The patient could express his or her attitude towards statements on a 6-point scale, where 1 denotes ‘strongly disagree’, and 6 ‘strongly agree’. The result range for each scale covers 6-36 points. The higher a scale results in, the stronger patient believes in a given dimension of health locus of control. This scale is accurate and reliable (13).

The AIS (Acceptance of Illness Scale) is applied to measure degree of acceptance of illness. It consists of 8 statements that describe negative consequences of undesirable health. The participants are asked to express their feeling by marking on each statement where 1 denotes ‘strongly agree’ and 5 ‘strongly disagree’. The higher a scale results in, the better patient accepts his/her illness and adapts more suitable to the illness with lower feeling of psychological discomfort. The AIS has satisfactory psychometric properties (13).

### 3.1. Statistical Analysis

Analyses were performed using the STATISTICA, 10th package. Correlation tests were used, including multiple regression analysis and tests of statistical significance of differences between mean results (Student’s t-test).

## 4. Results

Statistically, patients with Graves-Basedow disease differ significantly from those with Hashimoto disease in terms of health locus of control in other persons and increase in illness acceptance. However, there was no difference among the groups as to internal and external (i.e., the influence of a chance) health locus of control, (Table 1). Patients with Graves-Basedow disease more frequently had health locus of control in other persons and were less inclined to accept their illness compared to patients with Hashimoto disease.

**Table 1:** Significance of Differences Between Mean Results of Analyzed Variables in Both Patient Groups

	Graves-Basedow Disease (n = 68) a	Hashimoto Disease (n = 54) a	P value
Internal health locus of control	24.87 ± 5.19	24.87 ± 5.19	0.581
External health locus of control - Influence of other persons	29.65 ± 4.45	26.63 ± 5.14	0.001
External health locus of control - influence of a chance	23.62 ± 5.77	23.17 ± 5.42	0.660
Acceptance of the illness	28.79 ± 8.09	32.89 ± 7.56	0.005

^a^Mean ± SD

While performing analysis for both sexes, statistically significant differences were observed only between female patients in terms of two variables: external health locus of control in other persons, and illness acceptance (Table 2). Male patients in both groups did not differ in terms of analyzed variables. Female patients could not be compared with male patients due to too big discrepancy in patient population.

**Table 2:** Significance of Differences Between Mean Results of Analyzed Variables in Both Female Patient Groups

	Graves-Basedo Disease (n = 68) a	Hashimoto Disease (n = 54) a	P value
Internal health locus of control	24.67 ± 5.12	24.20 ± 5.22	0.645
External health locus of control - Influence of other persons	29.42 ± 5.36	26.59 ± 4.32	0.004
External health locus of control - influence of a chance	24.40 ± 5.28	23.43 ± 5.42	0.369
Acceptance of the illness	28.26 ± 7.58	32.91 ± 8.30	0.004

^a^Mean ± SD

In order to provide answer to research question No. 2 on relationship between health locus of control and illness acceptance with age, r-Pearson test was applied (Table 3).

**Table 3:** Significance of Correlation Between Analyzed Variables and Age in Groups G-B and H as Well as in Both Female and Male Patient Groups

	Graves-Basedow Disease	Hashimoto Disease	Women	Men
Internal health locus of control	0.128	-0.083	-0.008	0.181
External health locus of control - influence of other people	0.219	0.225	0.223 a	0.037
External health locus of control - influence of a chance	0.192	0.103	0.131	0.240
Acceptance of the illness	-0.170	-0.232	-0.114	-0.527 b

^a^P < 0.05

^b^P < 0.002

Analysis of entire patient groups showed a positive significant correlation (r = 0.193; P = 0.034) between age and external health locus of control in other people. The elderly patients, irrespective of the type of illness, were more inclined to think that the control of their health depended on others.

Analysis for each of the patient groups without age consideration did not reveal any statistically significant relationship between the age and beliefs in health locus of control and illness acceptance. Analysis for individual patient groups did not show, either, a statistically significant correlation between analyzed variables and the age. Yet, in analysis of patient groups when variable of their sex was taken into account, male patients with Graves-Basedow disease obtained a statistically significant result between the age and illness acceptance (r = -0.565; P = 0.044). The older they were, the more difficult it was for them to accept the disease. Other relationships were not statistically significant.

As to the response to research question No. 3 on relationship between health locus of control with illness acceptance in patients with Graves-Basedow and Hashimoto diseases, analysis were conducted only in female patient groups. Results obtained so far have indicated that the importance of sex-variable is substantial; male patient groups were quantitatively insufficient to be included in the analysis. Multiple regression analysis was applied.

Beliefs in health locus of control in the female patient group accounted for 14 % variability in illness acceptance (corrected R^2^ = 0.143; F (4.95) = 5.116 P = 0.0009); influence of beliefs was located in chance and the disease – either Graves-Basedow or Hashimoto disease – is very important (Table 4). When female patients suffering from Hashimoto disease had less frequently believed their health locus of control in chance, it was easier for them to accept their illness. Female patients with Graves-Basedow disease had more difficulties in accepting their illness.

**Table 4:** Results of Multiple Regression Analysis – a Disease and Beliefs in Health Locus of Control as Indicators of Acceptance of the Illness in the Female Patient Group

Variables Included in the Model	Standardized Regression Coefficient	Standard Error – z a	Regression Coefficient	Standard Error - z b	P value	Partial Correlation
Absolute term			32.111	7.500	0.000	.
Type of disease	0.223	0.097	3.677	1.609	0.025	0.228
Internal source of health control	0.147	0.103	0.235	0.164	0.156	0.145
External source of health control Influence of other peoplel	-.150	0.108	-0.247	0.178	0.169	-0.141
External source of health control Influence of a chance	-0.259	0.095	-0.399	0.147	0.008	-0.269

^a^Standardized regression coefficient

^b^Regression coefficient

## 5. Discussion

Currently, one can observe an explicit tendency to consider issues related to health and disease in a biopsychosocial context that takes into account the influence of psychological and social factors on the course of illness. The assessment of co-occurrence of these factors allows to explain health problems (21) more extensively than before.

In case of suffering from a chronic illness of the thyroid gland it is essential to adapt to it and thus the patient can face with this new and difficult situation. The adaptation is reflected, inter alia, in the degree of illness acceptance that can be an emotional indicator of living and functioning with the illness (12, 13). A question arises as to conditions for illness acceptance and as to whether its intensification is determined only by specific nature of the disease or maybe by personality features, e.g. beliefs in health locus of control. This article aims at finding the answer to this question.

The first stage of analysis was concerned with the question about the specificity of beliefs and illness acceptance depending on the type of disease. Results obtained indicate that patients with Graves-Basedow disease substantially frequently have their health locus of control in other people and are less inclined to accept their illness than patients with Hashimoto disease. A question emerges as to the course of the illness, which results in evoking a belief in the necessity to trust others and in difficulties in accepting the currently experienced situation. Should we look for the causes of this situation in personality features, or rather in the specific nature of the disease? Do both these factors perform an essential role? Research conducted so far (22) corroborate a substantial role of personality features, and neuroticism in particular, which does render adaptive functioning – both in health and in illness – really difficult. However, no differences in personality features between female patients and the control group were found; this refers both to female patients with Graves-Basedow disease and Hashimoto disease. Still, differences were observed in health-dependent personality features. Women with more severe course of the illness including ophthalmopathy tended to be more neurotic (23). In this article we are focusing on the importance of the specific nature of the illness as a differentiating element.

When we look at a chronic disease as a difficult situation (24) then possibly the course of Graves-Basedow disease is related to bigger difficulties (complications, ophthalmopathy, thyroid crisis) than the course of Hashimoto disease (25, 26). Moreover, the outward appearance changes more in Graves-Basedow disease than in Hashimoto disease, which is particularly important for women. For that reason it is not surprising that the analyzed difference occurs only between female patients. Other results also corroborate the fact that the increase in complications in the course of illness resulted in decrease in its acceptance and the increase in negative attitudes in patients with rheumatoid arthritis (15).

The correlation between health locus of control and acceptance of the illness, and the age of patients occurred to be important. Elderly female patients, irrespective of the type of disease, were more inclined to have health locus of control in other people. This result is in concord with results obtained so far indicating that the elderly patients are more inclined to give the control over their health into the hands of other persons (27). The elderly were convinced that their health control lies within self-confidence of others. It appears that with age, the importance of influence of other people on one’s own health tends to increase (13). In the control group this correlation is not that explicit, e.g. longitudinal studies conducted among the elderly (after 65 years of age) did not show any correlation between age and health locus of control (28). Probably these correlations look differently in the group of very old persons.

Yet, the individual with age, even if s/he is not chronically ill, more frequently may experience his or her own weakness and acknowledges the fact that owing to the assistance of others, a variety of issues in his or her life may take an encouraging turn. Social experience shows that the individual needs help offered by others, and this feeling is even stronger when time goes by. Such persons also respond more favourably to therapeutic treatment. For instance, cancer patients were observably less depressive when they had their external health locus of control in other people (29). Research conducted on various patient groups indicates more frequent external health locus of control, either in other people or in fate, chance, or luck, whereas internal health locus of control is at the level similar to that occurring in healthy controls. Are these results partly due to the disease? This question seems hard to be answered. No longitudinal studies were executed, and only comparative research of various patient groups could facilitate answering this question (22).

The primary and basic question in the research presented was concerned with the role performed by beliefs in health locus of control in the acceptance of illness. The multiple regression analysis revealed that in the female patient group, these beliefs can be treated as indicators of the illness acceptance, since taken together they account for ca. 14 % of cases of the illness acceptance variability. The most important part of this variability can be contributed to beliefs with their locus in chance as well as the type of disease (Table 4). When female patients suffered from the Hashimoto disease and less frequently had their health locus of control in chance, the easier it was for them to accept their illness. Female patients with Graves-Basedow disease as well as female patients with their health locus of control in chance had more difficulties in accepting their illness. External health locus of control is generally associated with negative emotions such as depression, hostility, anxiety and physical symptoms (11), thus it is not surprising that it makes living with the illness more difficult on the emotional level, i.e., accepting the illness.

The results should be treated with some caution, since they have been obtained by examining a small group of patients and a small group of men.

In summary we have Conclusions:

1. Research patients with Graves-Basedow disease substantially frequently have their health locus of control in other people and are less inclined to accept their illness than patients with Hashimoto disease. This correlation refers to female, not male patients.

2. A significant positive correlation between age and external health locus of control in other people was observed. The elderly patients, irrespective of the type of disease, were more inclined to think that controlling their own health depended on other people.

3. Male patients with Graves-Basedow disease, while getting older, found it more difficult to accept their illness.

4. Beliefs in health locus of control in the female patient group accounted for 14 % of variability of illness acceptance and could be treated as an indicator of illness acceptance. The most important contribution was made by beliefs in health locus of control in chance and the type of disease. When female patients suffered from Hashimoto disease and less frequently had their health locus of control in chance, they found it easier to accept their illness.
